# Diabetes and Colorectal Cancer Risk: A New Look at Molecular Mechanisms and Potential Role of Novel Antidiabetic Agents

**DOI:** 10.3390/ijms222212409

**Published:** 2021-11-17

**Authors:** Jelena Vekic, Aleksandra Zeljkovic, Aleksandra Stefanovic, Rosaria Vincenza Giglio, Marcello Ciaccio, Manfredi Rizzo

**Affiliations:** 1 Department of Medical Biochemistry, Faculty of Pharmacy, University of Belgrade, 11000 Belgrade, Serbia; jelena.vekic@pharmacy.bg.ac.rs (J.V.); aleksandra.zeljkovic@pharmacy.bg.ac.rs (A.Z.); alex@pharmacy.bg.ac.rs (A.S.); 2 Department of Biomedicine, Neuroscience and Advanced Diagnostics, University of Palermo, 90100 Palermo, Italy; giglio.rosaria.vincenza@gmail.com (R.V.G.); marcello.ciaccio@unipa.it (M.C.); 3 Department of Laboratory Medicine, University Hospital, 90100 Palermo, Italy; 4 Department of Health Promotion, Mother and Child Care, Internal Medicine and Medical Specialties, University of Palermo, 90100 Palermo, Italy

**Keywords:** insulin resistance, hyperglycemia, oxidative stress, inflammation, small dense LDL, glucagon-like peptide-1 receptor agonists

## Abstract

Epidemiological data have demonstrated a significant association between the presence of type 2 diabetes mellitus (T2DM) and the development of colorectal cancer (CRC). Chronic hyperglycemia, insulin resistance, oxidative stress, and inflammation, the processes inherent to T2DM, also play active roles in the onset and progression of CRC. Recently, small dense low-density lipoprotein (LDL) particles, a typical characteristic of diabetic dyslipidemia, emerged as another possible underlying link between T2DM and CRC. Growing evidence suggests that antidiabetic medications may have beneficial effects in CRC prevention. According to findings from a limited number of preclinical and clinical studies, glucagon-like peptide-1 receptor agonists (GLP-1RAs) could be a promising strategy in reducing the incidence of CRC in patients with diabetes. However, available findings are inconclusive, and further studies are required. In this review, novel evidence on molecular mechanisms linking T2DM with CRC development, progression, and survival will be discussed. In addition, the potential role of GLP-1RAs therapies in CRC prevention will also be evaluated.

## 1. Introduction

Diabetes mellitus (DM) is a complex metabolic disorder characterized by chronic hyperglycemia due to inadequate insulin secretion and/or insulin resistance [1]. Currently, DM is considered one of the largest epidemics in the world [2]. The increasing trend of newly-diagnosed patients illustrates the need to improve diabetes awareness and the prevention of its adverse consequences. Besides microvascular and macrovascular complications, DM has also been associated with increased risk for the development of different types of cancers, particularly endocrine and gastrointestinal malignancies [3]. Although both type 1 (T1DM) and type 2 diabetes mellitus (T2DM) patients are prone to cancer development, more evidence is available for T2DM, so the current review is focused on this specific population.

Colorectal cancer (CRC) is amongst the most frequently diagnosed cancers worldwide [4]. A strong epidemiological link between T2DM and CRC was recently confirmed in a meta-analysis of 150 studies, including data on 32 million people [5]. One of the challenges in CRC prevention among patients with T2DM is the complex etiopathological relationship between the two traits. Namely, T2DM and CRC share several common risk factors, such as a sedentary lifestyle, the Western pattern diet, obesity, and metabolic syndrome [6]. Evidence suggests that T2DM could also be causally related to CRC development through the effects of chronic hyperglycemia and hyperinsulinemia [7]. In addition to CRC risk augmentation, pre-existing T2DM is one of the factors that may affect patients’ response to neoadjuvant chemotherapy [8]. Furthermore, diabetes has been shown to increase the risk of CRC recurrence [9]. According to the meta-analysis of Zhu et al. (2017) [10], colorectal, colon, and rectal cancer patients with diabetes had a 5-year shorter survival compared to patients without diabetes.

Research on the incidence of metabolic disorders in CRC survivors is generally limited, but recent studies suggest an increased risk of diabetes among CRC survivors [11]. Singh et al. [12] showed that the risk for developing T2DM within 5 years of initial CRC diagnosis is consistent for both colon and rectal cancer patients. A recent prospective study including more than 7000 CRC survivors showed a significantly higher risk of T2DM with complications as compared to the general population [13]. These data indicate that a hazardous link between T2DM and CRC is bidirectional.

According to joint recommendations of the American Diabetes Association and American Cancer Society, patients with diabetes are identified as a high-risk group that should be targeted for regular cancer screening [14]. The implementation of early CRC screening in diabetes patients has been recently evaluated in a meta-analysis of 18 studies by Bhatia et al. [15]. Although the authors found comparable CRC screening rates between individuals with and without diabetes, women with diabetes were less likely to be screened compared to diabetes-free women [15]. The question of whether diabetes treatment might modulate the risk of cancer or affect its prognosis is an ongoing debate. So far, the most convincing evidence in reducing the incidence and improving the outcome of CRC has been reported for metformin [16]. In contrast, insulin users have shorter cancer-specific and overall survival [17]. Therefore, expert societies advise risk/benefit analysis in the selection of antidiabetic medications for patients with very high risk for cancer or cancer recurrence [14]. However, there is sparse data on the possible impact of novel antidiabetic therapeutics, such as glucagon-like peptide-1 receptor agonists (GLP-1RAs), on the risk for CRC development and recurrence. In this review, the link between T2DM and CRC will be evaluated through the contribution of insulin resistance, hyperglycemia, oxidative stress, and inflammation. The potential impact of novel antidiabetic drugs in CRC prevention will also be discussed.

## 2. Molecular Mechanisms Linking Diabetes and Colorectal Cancer

### 2.1. Insulin Resistance

Insulin resistance (IR) is probably the most obvious link between T2DM and CRC since both diseases are reportedly associated with this metabolic disorder. While the pivotal role of IR in the pathogenesis of T2DM is well known, currently, a bulk of data implicates the involvement of various aspects of IR in colorectal carcinogenesis. Ever since the 1990s, the “hyperinsulinemia-cancer of colon” hypothesis has been endorsed [18].

IR was seldom investigated as an isolated pathophysiological phenomenon in epidemiological studies of CRC, but many previous studies pointed towards the contribution of obesity and metabolic syndrome (MS) to the increased risk for CRC development [19,20,21]. Available evidence suggests that this association is gender-specific and stronger in men [22,23]. Since IR is a cornerstone of MS and obesity, it is assumed that IR and its specific metabolic disarrangements can play an active role during the onset and progression of CRC. Indeed, a recently published large cohort study of Okamura et al. [24] demonstrated that triglyceride-glucose index, as a marker of IR, is an independent predictor of CRC onset. Furthermore, a meta-analysis has revealed that the homeostasis model of risk assessment-insulin resistance (HOMA-IR) was significantly associated with CRC risk [25]. In addition, a recent Mendelian randomization study pointed towards a potential causal relationship between genetically driven IR and CRC risk in postmenopausal women [26]. However, it should be noted that a retrospective study that used the National Health and Nutrition Examination Survey (NHANES) data [27] failed to demonstrate a significant difference in HOMA-IR between patients with and without CRC within a time frame of 5 years. However, it should be noted that such results might arise due to cancer-related cachexia and possible weight loss during CRC treatment.

**Figure 1:**
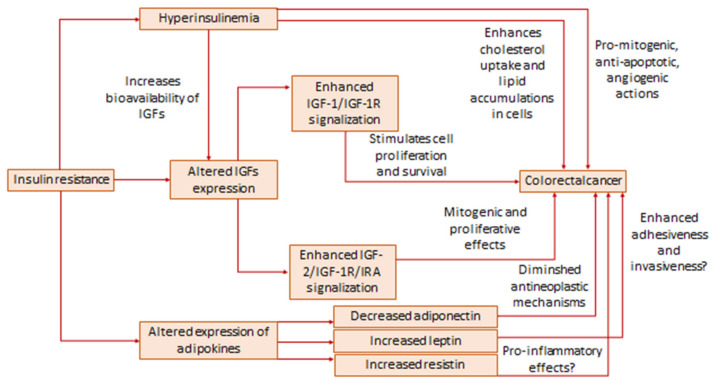
The role of insulin resistance in pathogenesis of CRC.

Several mechanisms have been proposed regarding the involvement of IR in the pathogenesis of CRC (Figure 1). First of all, hyperinsulinemia is a hallmark of IR, and it is well established that insulin contributes to cancer development by stimulating pro-mitogenic, angiogenic, and anti-apoptotic effects, mainly through binding to insulin receptor A, which is expressed in cancer cells [28,29]. It has been demonstrated that insulin stimulates proliferation of Caco-2 and HT-29 cell lines [30] but also enhances cholesterol uptake and lipid accumulation in Caco-2 cells through elevation of cholesterol transporter scavenger receptor, class B, type I (SR-BI) mRNA, and protein levels [31]. This latter effect of insulin might be of special interest, noting the increasing demand of malignant cells for cholesterol and evidence of disturbed lipid homeostasis in CRC [32,33]. Molecular mechanisms that directly link insulin action and the onset and progression of CRC are suggested by a number of studies. It was shown in a study on Caco-2 and HT-29 cell lines [34] that insulin induces c-Myc expression by using both mTOR and Wnt/β-catenin signaling pathways. On the other hand, insulin is a well-known inhibitor of glycogen synthase kinase-3β (GSK-3β), and it has been shown that downregulation of GSK-3β potentiates carcinogenesis through activation of Wnt/β-catenin signaling [35,36]. Additionally, indirect effects of insulin via insulin-like growth factors (IGFs) during cancer progression should also be taken into account. Surprising recent findings are related to the potential role of insulin in the enhancement of chemotherapeutics’ effects. Namely, it has been shown that pretreatment of Caco-2 and SW480 cells by insulin increases their susceptibility to the effects of anticancer therapy, and such effect is likely achieved by the downregulation of the phosphatidyl inositol 3-kinase (PIK3) pathway [37]. Of note, IR is typically associated with poorer response to anti-CRC therapy [28], and such novel findings might shed light on possible dose- and time-dependent effects of insulin on important molecular pathways of carcinogenesis.

Apart from hyperinsulinemia, IR is characterized by altered activities of IGFs. Insulin-like growth factor 1 (IGF-1) is the most studied insulin-like peptide in terms of its function in obesity, MS, IR, and cancer. In contrast to the increased insulin levels, IR is reportedly associated with decreased IGF-1 concentration, while therapeutic interventions have been shown to cause an increase of IGF-1 in IR subjects [38,39]. On the other hand, data on IGF-1 expression in CRC are ambiguous. While a number of studies implicate increased IGF-1 in CRC patients or a positive association of elevated IGF-1 with CRC risk [40,41,42], others demonstrated lower levels of this peptide in CRC [43,44,45]. However, it should be noted that CRC-induced cachexia and consequent impaired liver synthetic function could be the reasons for decreased IGF-1 levels. Nevertheless, it has been shown that IGF-1 receptor (IGF-1R) is overexpressed in CRC [28,46] and it is well established that the association of IGF-1 with its receptor stimulates cell proliferation and survival thus contributing to cancer development [28]. In addition, it was demonstrated that insulin receptor substrate-1, as the principal cellular effector of IGF-1R, is also overexpressed in CRC tissue [47,48], although a recent study by Lomperta et al. [49] suggested a possibility of its divergent roles in the regulation of apoptosis. Furthermore, activation of the IGF-1/IGF-1R pathway can be at least partly responsible for CRC treatment resistance [28]. The other insulin-like peptide, insulin-like growth factor 2 (IGF-2), although less investigated in comparison to IGF-1, is now considered a highly important contributor to CRC development. As recently reviewed by Kasprzak and Adamek [50], IGF-2 is involved in colorectal carcinogenesis by its mitogenic and proliferative effects, achieved mainly through the interaction with insulin receptor A and IGF-1R. IR can contribute to the enhanced effects of IGFs since hyperinsulinemia suppresses levels of IGF binding proteins (IGFBPs), thus increasing the bioavailability of IGFs [51]. However, such inhibitory effects of insulin on IGFBPs should not be observed as straightforward, since novel data indicate that IGFBPs themselves exhibit direct cellular effects, independently of IGFs, although so far conducted studies yielded mainly inconclusive results regarding the role of IGFBPs in CRC [42,52,53,54].

Contemporary scientific researches proposed genetic, epigenetic, and environmental factors as determinants of the biological effects of insulin-like peptides in various pathophysiological conditions. An interesting study by Hu et al. [40] indicated that IGF-1 gene expression levels were higher in patients with concomitant MS and CRC than in patients with CRC without MS. In addition, it has been demonstrated that specific single nucleotide polymorphisms (SNPs) of genes within IGF-1/IR traits, in interaction with modifiable lifestyle factors, affect CRC risk in postmenopausal women [55]. Moreover, a possible synergistic effect on CRC risk in postmenopausal women was reported for IR-associated SNPs LINC00460 rs1725459 and MTRR rs722025 in combination with estrogen/oral contraceptive use and smoking [56]. Both LINC00460, i.e., long intergenic non-coding RNA 460, and MTRR, which encodes methionine synthase reductase, are previously recognized as related to IR [56]. Finally, a novel approach to the analysis of shared genetic and epigenetic traits of IR and CRC includes microRNAs (miRNAs). It has been recently shown that dysregulation of various miRNAs is common for both IR and CRC [57]. Recently, it has been shown that targeted reduction of IGF-1R by let-7e miRNA ameliorates the sensitivity of CRC cells to radiotherapy [58]. An interesting new perspective in the analysis of possible associations of IR and CRC is opened by a hypothesis according to which vitamin D antagonizes pro-cancerogenic effects of IGF-1 [59]. It is well known that obesity, as the most prominent anthropometric marker of IR, is characterized by vitamin D deficiency [60]. Furthermore, vitamin D status is decreased, and vitamin D metabolic balance is disturbed in CRC [61,62], so IR can represent an additional link between reduced vitamin D level and increased risk for CRC.

Besides dysregulation of the insulin/IGFs axis, changes in adipokines are also inherent to IR. Altered levels of adipokines are reported in CRC, whereas adiponectin, leptin, and resistin are most often analyzed. Unlike the majority of adipokines which participate in the development of obesity-related complications, adiponectin possesses anti-inflammatory properties and improves insulin sensitivity and lipid status, thus acting protectively in maintaining metabolic homeostasis [60]. It was also demonstrated that adiponectin has antineoplastic properties [63]. Decreased adiponectin plasma levels are associated with both IR and CRC [64,65,66]. It has been shown that specific SNPs rs2241766 and rs1501299 of adiponectin encoding gene *ADIPOQ* are related to an increased CRC risk [67]. The mentioned SNPs are associated with decreased adiponectin levels and a higher degree of IR as well. However, it should be noted that other studies revealed the opposite results, namely increased levels of adiponectin in CRC or the lack of any relationships between adiponectin and CRC development [68,69,70]. Leptin participates in the control of energy expenditure and is considered a pro-inflammatory adipokine [60]. Hyperleptinemia and leptin resistance can trigger hyperinsulinemia and vice versa [71]. In addition, recent evidence indicates the role of leptin in the development of CRC. In particular, overexpression of leptin was observed in CRC tissue [72], and elevated leptin was recognized as an indicator of poorer CRC prognosis [73]. It has been shown that leptin ameliorates the adhesiveness and invasiveness of CRC stem cells [74]. In contrast, negative or variable associations between leptin plasma levels and CRC were also reported [70]. So far, the studies have yielded inconsistent results regarding characteristic SNPs of the leptin gene and CRC development [68,75]. Resistin, primarily produced by monocytes and macrophages, is another connecting point for IR and CRC, although the role of resistin in both pathophysiological processes is not fully explained. Resistin was reportedly increased in subjects with rectal cancer and in correlation with insulin levels in CRC patients [76]. Similarly, our study demonstrated increased levels of resistin and its receptor adenylate cyclase-associated protein 1 in CRC [77]. Also, a meta-analysis pointed towards elevated resistin levels in CRC patients [78]. On the other hand, resistin is considered a contributing factor to IR [79], thereby linking these conditions. However, having in mind incompletely understood mechanisms of resistin actions during both IR and CRC development, future studies are warranted in this area.

### 2.2. Hyperglycemia

Prospective data from the Malmö Diet and Cancer cardiovascular cohort, including 5144 subjects, demonstrated that high blood glucose level increases the probability of colon cancer development [80]. The authors also suggested sex-specific and tumor site-specific associations between hyperglycemia and CRC risk. In particular, the associations were significant in the subgroups of men and colon cancer patients but not in women and patients with rectal cancer, respectively [80]. In the study of Siddiqui and co-workers [81], patients with poorly controlled T2DM had more right-sided tumors, which usually comprise a poorer prognosis [82], more advanced forms of the disease, as well as shorter survival. The subsequent study of Lee et al. [83] confirmed that poorly controlled DM is associated with a higher risk of mortality and shorter survival. Based on the study results, the authors [83] suggested maintaining the optimal glycohemoglobin (HbA_1c_) level of ≤7.8% in CRC patients with DM. 

Proliferating malignant tumor cells have high energy turnover and greater glucose demand [84]. In line with this, increased expression of GLUT1 was reported in various malignant tissues, as well as in CRC [85]. According to Yang et al. [86], GLUT1 overexpression in CRC tissue could be considered as an indicator of tumor aggressiveness and poor prognosis. In the setting of hyperglycemia, the growth of CRC tissue is further accelerated [87]. Poor glycemic control in diabetes activates polyol and hexosamine metabolic pathways [88], and both might be involved in colorectal carcinogenesis and metastasis. The evidence suggests that fructose, produced in the polyol pathway, might accelerate cancer growth [89]. Overexpression of the polyol pathway enzymes, sorbitol-dehydrogenase, and aldose-reductase, have been found in colorectal adenomas [90] and colon cancer cells [91]. Furthermore, inhibition of aldose-reductase, the rate-limiting enzyme of the polyol pathway, prevented invasion and migration of cultured human colon cancer cells [92]. Regarding the hexosamine pathway, it was demonstrated that its endproduct, UDP-β-D-N-acetylglucosamine (UDP-GlcNAc) [88], plays an important role in cancer development. It is able to induce the O-GlcNAcylation process, a post-translational modification of serine and/or threonine residues of different cytoplasmic proteins, which further affects various cell functions, including cell cycle, chromatin dynamics, gene expression, and cell adhesion [93]. In line with previous data, O-GlcNAcylation was also found to be increased in colon cancer cells, particularly in those with higher metastatic potential [94]. Protein kinase C activation is another putative mechanism by which hyperglycemia contributes to CRC development and progression. Namely, hyperglycemia is followed by a persistent increase of diacylglycerol, which stimulates the activity of the classic protein kinase C isoforms α, β, and δ [88]. As reported in studies on colon carcinoma cells and experimental models of colon cancer, activation of PKC α and β isoforms is involved in carcinogen-induced malignant transformation, proliferation, migration, and survival of colon cancer cells [95,96]. 

Another important aspect of hyperglycemia is related to the chemoresistance of malignant CRC cells and the development of metastases. Ma et al. [97] demonstrated that high glucose level increases CRC cell resistance to 5-fluorouracil. Ikemura and colleagues [98] showed that hyperglycemia is associated with diminished effects of 5-fluorouracil and oxaliplatin treatment and reduced survival in an animal model of CRC. More recently, it was confirmed that hyperglycemia per se is associated with poor outcomes of stage III CRC patients treated by FOLFOX adjuvant chemotherapy, including 5-fluorouracil/folinic acid and oxaliplatin [99]. The same study also provided evidence that chemoresistance of CRC cell lines was reversed following the administration of glucose-lowering agent metformin [99]. 

**Figure 2:**
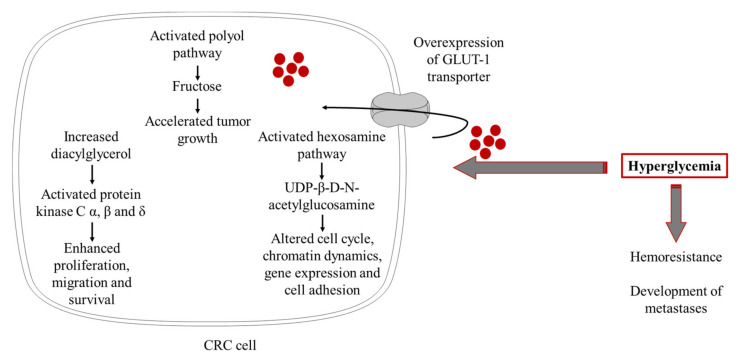
The role of hyperglycemia in pathogenesis of CRC (The figure was composed using Servier Medical Art templates, licensed under a Creative Common Attribution 3.0 https://smart.servier.com (accessed on 12 November 2021)).

Overall, data from in vitro and in vivo studies support a causal role of hyperglycemia in CRC development and progression (Figure 2). Therefore, maintenance of optimal glycemic control seems to be a promising strategy for both T2DM and CRC prevention. Taking into account recent achievements in the management of diabetes by introducing novel glucose-lowering medications with beneficial effects on cardiovascular risk, future studies demonstrating the role of emerging diabetes therapies in CRC prevention are warranted.

### 2.3. Oxidative Stress and Inflammation

**Figure 3:**
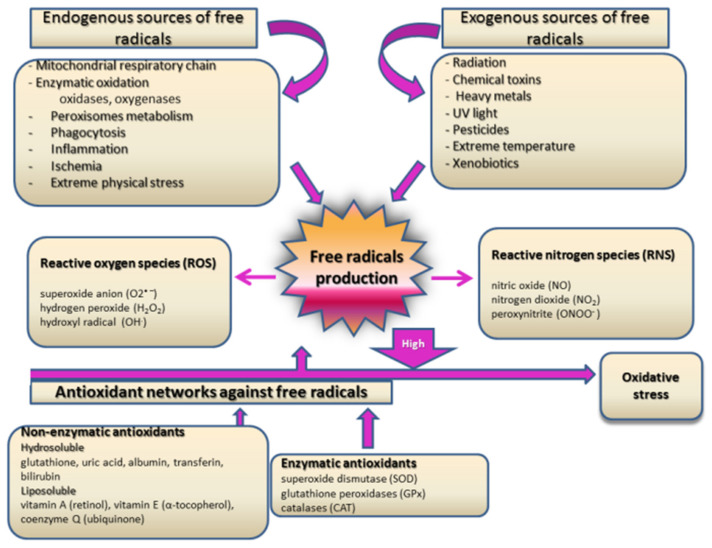
Major sources of free radicals and main antioxidants.

Among many different possible molecular mechanisms involved in the complex etiology of T2DM and CRC, multifactorial risk assessment singled out synergistic effects of oxidative stress and inflammation as crucial components for the pathogenesis of both diseases [100]. Oxidative stress is defined as an imbalance between free radicals production, especially reactive oxygen species (ROS), and available antioxidative capacity for their detoxification [101] (Figure 3). In specific physiological and pathological conditions, when increased free radicals production cannot be completely neutralized by enzymatic and non-enzymatic antioxidants, proteins, lipids, nucleic acid DNA, and some cellular components are oxidatively damaged [102]. Resolving the molecular reaction to increased oxidative stress and physiological and pathological cellular responses to such stimuli is essential to better understand the synergistic effects of different diseases’ pathogenesis.

Research in the past few decades proved that oxidative stress plays a major role in the onset of diabetes and the development of its complications [100,103,104,105,106,107]. However, mechanisms by which oxidative stress contributes to those processes remain a subject of continual debate. Hyperglycemia and increased total plasma-free fatty acid concentration are the main features of DM [104]. Hyperglycemia leads to increased ROS production in mitochondria and undoubtedly to intense oxidative stress by processes of non-enzymatic proteins glycation and glucose autooxidation [104]. As we explained previously, hyperglycemia directly activates different molecular pathways such as glycolysis, advanced glycation endproducts (AGEs) formation, protein kinase C activation, hexosamine, and polyol pathways [105]. All those specific molecular processes encompass hyperglycemia-induced oxidative stress as a unifying mechanism [105]. Increased concentrations of free fatty acids boost acetyl-CoA production that feeds into the Krebs cycle, making excess NADH and more superoxide anion generation and, consequently, cyclic acceleration of oxidative stress [106]. Oxidative stress is also responsible for antioxidant enzyme deactivation and reduced glutathione, which multiply the excessiveness of oxidative damage [108]. The main aspects of oxidative damage in diabetes and its associated secondary complications are manifested as protein oxidation [109], lipid peroxidation [110], and DNA oxidation, which might be directly linked to enhanced cancer risk in diabetic patients [111,112,113].

There is growing support for the theory that oxidative stress, especially oxidatively modified DNA and lipid peroxidation products, has a crucial role in the pathogenesis of CRC [114,115,116,117]. In light of the fact that there is a considerable need for new diagnostic and prognostic biomarkers for cancer pathogenesis, results of some investigations highlight 8-hydroxy-2’-deoxyguanosine (8-OHdG) as a potent additional tumor marker [114,115]. Matosevic et al. [118] reported a higher level of 8-OHdG in tissue samples of colorectal carcinoma than in healthy mucosa cells. Recently, Abudawood et al. [113] revealed significant interconnections between 8-OHdG levels and cancer biomarkers, carcinoembryonic antigen (CEA), and CA 19-9 concentrations in T2DM patients, supporting the concept that the diabetic environment could be a source of oxidative stress and consequently could lead to cancer development. Kang et al. [119] demonstrated that DNA damage generates oxidative stress per se by H2AX-Nox1/Rac1 pathway activation, induces cell apoptosis, and accelerates oxidative damage. Furthermore, Kitagawa et al. [120] suggested a ratio of 8 OHdG in DNA and 8 OHdG in the cytoplasm as a marker for predicting cancer recurrence and post-operative survival among patients with CRC, thereby increasing the diagnostic value of this oxidative biomarker in CRC. 

According to previous research, the products of lipid peroxidation might have an important role in carcinogenesis and serve as potential therapeutic targets in CRC patients [121,122]. It seems that malondialdehyde and 4-hydroxy-2-nonenal, as endproducts of lipid peroxidation, have a great capability for laboratory evaluation of CRC, in spite of their lack of specificity for cancer [123,124]. Recently, Janion et al. [125] found that the intensity of lipid peroxidation in CRC patients is associated with primary tumor location. Their results showed that higher malondialdehyde concentrations in CRC patients are linked with right-sided or distal tumor location, which has a poorer disease prognosis [125]. It has been demonstrated that 4-hydroxy-2-nonenal induces cyclooxygenase-2 (COX-2) expression [126] and is directly associated with colorectal carcinogenesis [127] through the promotion of prostaglandin E2 biosynthesis, which increases the proliferation and invasiveness of epithelial cancer cells [128]. COX-2 is recognized as one of the major catalysts for the progression of CRC. Numerous clinical trials have investigated COX-2 as a molecular target for CRC therapy in terms of the introduction of non-steroidal anti-inflammatory drugs into standard therapeutic protocols and also as a potent chemopreventive agent in the high-risk population [129]. However, there is a growing need for further investigations in this field for more decisive conclusions.

Another aspect of the proposed mechanisms for the involvement of oxidative stress in the development of different diseases, in particular diabetes progression and cancer development, is the fact that free radicals are modulators of cellular signaling pathways and, thereby, regulators of different cellular functions such as proliferation, growth, and apoptosis [130]. It is well accepted that oxidative stress induces specific stress-activated protein kinase and p38 reactivating kinase pathways, and these processes result in modifications of cellular gene expression and increase inflammation [131]. Furthermore, ROS stimulates potent nuclear factor-kappa B (NF-κB), a transcription factor that regulates the synthesis of pro-inflammatory cytokines and adhesion molecules. In turn, pro-inflammatory cytokines induce low-grade inflammation and indirectly provoke oxidative stress [132]. Their complex interplay creates vicious circles that induce further oxidative damage and multiple activations of previously mentioned stress-induced cellular pathways. Transcription factor NF-κB also regulates the expression of genes involved in cell proliferation, apoptosis, and differentiation, which indicates that its activation has a potential role in malignant transformation of cells and tumor progression [133]. Indeed, activation of NF-κB is a firmly established mechanism of CRC development and progression [134]. Increased production of pro-inflammatory cytokines interleukin 6 (IL-6), tumor necrosis factor-alpha (TNF-α), and IL-1 [135] have been observed to activate Akt and Wnt, two signaling pathways that have also been implicated in CRC carcinogenesis [136]. Furthermore, recent data linked the activation of the NOD-like receptor pyrin domain 3 (NLRP3) inflammasome with the development of certain types of cancers, including CRC [137]. In line with this, activation of NLRP3 by high glucose levels was demonstrated by Wang et al. [138]. Shi and colleagues [139] recently showed significantly upregulated expression of NLRP3 in colon adenocarcinoma tissues as compared to adjacent normal tissue. In addition, high expression of NLRP3 was associated with poor prognosis and reduced survival of colon cancer patients [139]. The main role of inflammasomes is the regulation of caspase-1 activity, which promotes the secretion of pro-inflammatory cytokines IL-1β and IL-18. So far, numerous studies have confirmed that IL-1β is an important player in intestinal inflammation [140], as well as its role in tumorigenesis and invasiveness of CRC [141]. 

It was also hypothesized that oxidative modification of proteins stimulates carcinogenic pathways [142,143]. Advanced oxidation protein products (AOPPs) and advanced glycation end products (AGEs), which are excessively generated in hyperglycemia and oxidative stress, are the most investigated indicators of oxidative protein modifications in diabetes and CRC [144]. Higher expression of AGEs is found in colon cancer tissue as compared to adjacent normal mucosa [145]. Both AOPPs and AGEs are able to interact with receptors for advanced glycation end products (RAGE), which triggers initiation of mitogen-activated protein kinases (MAPK), p38, PKC, and NF-κB signal transduction cascades [146,147]. Ample evidence indicates that RAGE dysregulation could be involved in the pathogenesis of different malignancies, including CRC [148,149]. RAGE stimulation induces inflammation, disables cell apoptosis, and stimulates angiogenesis, thus leading to the generation of a specific oxidative/inflammatory milieu, which favors tumor growth and progression [150]. Interestingly, recent data from the European Prospective Investigation into Cancer and Nutrition (EPIC) study showed an inverse association between plasma levels of several methylglyoxal and glyoxal-derived AGE species and CRC risk [151]. These findings indicate that measurement of circulating AGEs is likely insufficient to monitor CRC risk and activity since it does not reflect AGEs accumulation in situ [151]. On the other side, EPIC study researchers also showed that pre-diagnostic circulating sRAGE levels are inversely associated with CRC risk, at least in men [152]. Circulating sRAGE-AGE complexes prevent tissue accumulation of AGEs and their consequent detrimental effects [153]. However, whether sRAGE could be a useful diagnostic and prognostic biomarker for CRC remains to be established in the future.

However, complex redox biology in CRC needs to be considered in light of free radicals generation as a consequence of anticancer therapy, too [116,121]. Almost all conventional cancer therapies (chemotherapy, radiotherapy) are based on free radicals’ production [116,121,154,155,156,157]. Lee et al. [156] proved that tamoxifen induces cellular senescence as a tumor suppression mechanism in colon cancer by decreasing the catalytic activity of protein kinase CK2 through intense ROS production. Radiotherapy causes cellular stress followed by free radicals production, which leads to the death of malignant cells [157]. Molecular mechanisms of free radicals function in cancer therapy can be interpreted in two different ways [158]. The first aspect is reflected in reducing oxidative stress and suppressing oxidative stress-induced cellular signaling pathways to decrease oxidative cell damage and malignant transformation. This approach stresses the importance of antioxidants in chemoprevention and cancer treatment. The second aspect is based on the evidence that higher induction of oxidative stress in cancer cells with already impaired antioxidative capacity accelerates cellular death, and this mechanism has been established as a potential tool in cancer therapy [116,121,154,155,156,157,158]. Both aspects investigate the precise mechanisms that underlie the role of oxidative stress in cancer initiation and progression, and both are a bountiful area of scientific interest for future molecular antidiabetic and anticancer therapeutic approaches. 

## 3. Novel Antidiabetic Glucagon-like Peptide-1 Receptor Agonists (GLP-1RAs) Therapies and Colorectal Cancer

In recent years, the development of new hypoglycemic drugs has significantly enriched the therapeutic options available for the management of T2DM [159]. Among these novel treatments, glucagon-like peptide-1 receptor agonists (GLP-1RAs) mimic the effects of human native GLP-1 that, produced in enteroendocrine cells and secreted after meal ingestion, regulate glycemia through glucagon inhibition and stimulation of insulin secretion by pancreatic beta cells in a glucose-dependent manner [160]. In diabetic subjects, the incretin effect is reduced or absent; GLP1-RAs are able to restore this balance and are therefore widely used for the treatment of T2DM due to their ability to lower glucose and cardiometabolic risk, with a low risk of hypoglycemia [161]. The most frequent side effects are nausea and other minor gastrointestinal symptoms, which are usually transient and mild in intensity, mainly occurring in the dose-escalation phase of the treatment [162]. 

There is currently interest in GLP-1RAs therapies used to treat T2DM patients for the growth of cancer cells. It has been shown that incretin mimetic therapy may act as a promoter of the development of pre-existing precancerous lesions and metaplasia of the pancreas, thus increasing the likelihood of progression to pancreatic cancer [163]. An association of GLP-1RAs with the onset of pancreatic cancer has been suggested [164], although subsequent experience from clinical practice globally has revealed that the frequency of this neoplasm is quite rare with the use of GLP-1RAs. A very recent analysis of real-world databases evaluated the risk of various cancers among GLP-1RAs recipients in relation to the use of metformin, the most widely used traditional antidiabetic therapy. The authors were able to identify over 600,000 and 60,000 patients in the metformin and GLP1-RA groups, respectively [165]. In relation to metformin, treatment with GLP1-RAs was associated with a lower risk of prostate, lung, and colon cancer, while the risk of thyroid cancer was higher. In addition, the risk of prostate, lung, and colon cancer further decreased with a longer duration of GLP1-RA use, suggesting an exposure duration-response relationship [165].

This finding of a potential adverse GLP1-RAs action on thyroid cancer is somewhat consistent with previous findings from clinical trials. Indeed, thyroid adverse events, including increased plasma calcitonin, goiter, and thyroid neoplasia, have been mostly seen in patients with pre-existing thyroid disease, although with rare frequency [166]. GLP-1 also exerts independent effects that may promote cell growth and survival, and prolonged activation of the GLP-1 receptor signal in rodent thyroid glands is known to lead to C-cell hyperplasia and medullary thyroid cancer [167]. By contrast, it is of great interest that consistent data are emerging on the beneficial effects of GLP1-RAs on colon and breast cancer. This class of drugs seems not to affect the growth or survival of human colon cancer cells and may be therefore safe for diabetic colon cancer patients. In addition, the longer GLP1-Ras are used, the longer the benefit is on colon cancer [165].

These aforementioned clinical data supporting a protective effect of GLP1-RAs on colon cancer are also somewhat supported by existing evidence from preclinical studies [168]. This class of drugs did not accelerate malignancy in mice treated with carcinogens and did not modify the growth or apoptosis of human colon cancer cells [169,170], while in another study, GLP1-RAs inhibited growth and augmented apoptosis in murine colon cancer cells [171]. Consistent with that report, antidiabetic drugs that stabilize incretin hormones are known to affect colon carcinogenesis, and such protective effects could be exploited in studies of chemo-prevention [172]. However, it has also been reported that GLP1-RAs may promote pathways with a potential role in the development of colorectal cancer [173], which supports the need for further studies in order to reach a definitive conclusion on the role of these antidiabetic agents on colon cancer. Indeed, we cannot consider available data from the aforementioned clinical and preclinical studies as conclusive due to methodological limitations of the analyses, such as the low number of samples evaluated and a number of potential sources of bias, including differences between studied groups with respect to age, gender, disease duration, and concomitant treatments. 

**Figure 4:**
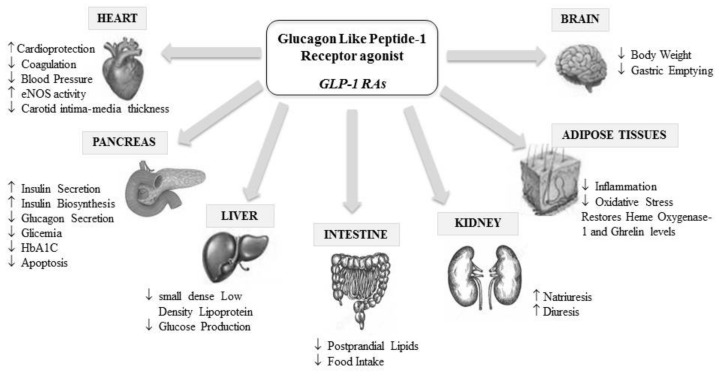
Glucagon-like peptide-1 receptor agonist (GLP-1RAs) pleiotropic effects.

GLP1-RAs also have a positive cardiovascular outcome in T2DM patients, reducing cardiovascular events and mortality, which can be explained by their anti-inflammatory and anti-atherogenic properties [174]; indeed, these agents have a number of important extra-pancreatic cardiometabolic beneficial actions, as summarized in Figure 4 [175,176].

Regarding plasma lipids, GLP1-RAs are able to improve both the quantity and the quality of low-density lipoproteins (LDL) by reducing levels of denser LDL with smaller size [177,178] in contrast to other antidiabetic agents that have a null or negative effect on such particles [179]. These subspecies are highly atherogenic due to reduced LDL-receptor affinity, higher susceptibility to oxidation, and greater arterial entry and retention [180]. Indeed, small, dense LDL becomes quickly modified in oxidized particles (ox-LDL) inside the intima, particularly when oxidative stress is higher. This triggers the formation and stratification of foam cells in the vessel wall, representing the first step of the atherosclerotic cascade, which is also enhanced in the case of endothelial dysfunction and inflammation [181]. Therefore, the predominance of small, dense LDL represents a critical factor for the development and progression of atherosclerosis [182] and endothelial dysfunction, which both amplify the risk of cardiovascular events [181]. Some inflammatory cytokines, such as resistin, are closely associated with these LDL subspecies and seem to play a role in both diabetes and colon cancer development [77,183]. 

Interestingly, patients with colon cancer have elevated levels of small dense LDL [184], and this is in line with previous findings showing that higher concentration of ox-LDL may participate in colon cancer development, probably by binding to oxidized LDL receptor that significantly contributes to the transformation, cell motility, and growth of cancer cell lines [185,186]. In addition, since cancer cells overexpress LDL receptors, LDL particles are significantly absorbed by these cells [184]. This is also facilitated by the fact that small dense LDL particles have a lower affinity for LDL receptors and therefore remain for a longer time in the plasma [180]. Finally, another specific property of small dense LDL is increased susceptibility to oxidation when compared to larger LDL counterparts, thus creating oxLDL, which may be involved in the development of cancer. T2DM patients are particularly prone to develop oxidative stress and atherogenic lipoprotein phenotype [180], comprising small dense LDL particles, increased triglycerides, and reduced HDL-cholesterol levels, and such lipid status perturbations might represent another possible underlying link between T2DM and cancer.

At present, numerous case-control and retrospective cohort studies have evaluated the potential effects of T2DM therapy on CRC risk reduction. On the other side, a limited number of clinical trials have been conducted to specifically address the impact of antidiabetic medications on the risk for CRC development or survival of diabetic patients with CRC (Table 1). In line with the fact that metformin is the most commonly used hypoglycemic agent, several clinical trials reported its chemopreventive effect against CRC development in non-diabetic subjects [187,188,189]. Two clinical trials in T2DM patients, ADOPT and RECORD trial, recorded incidences of various malignancies during the follow-up period and provided valuable data to study the potential impact of metformin, rosiglitazone, and sulfonylurea derivatives therapy on CRC risk [190]. Based on these data, it appears that metformin and rosiglitazone have similar effects, but sulfonylurea derivatives, such as glyburide (i.e., glibenclamide), should be considered with caution [190]. According to the results of the LEADER trial, GLP1-RA liraglutide did not increase the incidence rate of colon and rectal cancers, as compared to placebo [191]. Furthermore, available evidence from clinical trials in CRC patients with T2DM indicates that metformin use has no significant impact on patients’ survival [192,193]. However, the number of included participants was rather small, and the follow-up period was relatively short to yield a firm conclusion. As far as we are aware, no clinical trials have evaluated a specific association between other antidiabetic medications and survival of CRC patients with T2DM.

At this point, the mechanisms that link mentioned antidiabetic therapeutics with CRC should be briefly discussed. The role of insulin in CRC development was already explained and has also been confirmed in clinical trials [194]. Similarly, treatment with sulfonylurea derivatives, such as glibenclamide, might increase susceptibility for CRC development [190,195]. The plausible mechanism which associates glibenclamide with CRC could be enhanced insulin secretion and consequent activation of the IGF-1–IGF-1R axis [196]. On the other side, metformin has been reported to have chemopreventive potential against CRC, mainly by its effects on AMP-kinase activity and subsequent inhibition of IGF-1 and mTOR signaling pathways [197]. Next, rosiglitazone demonstrated anti-proliferative and pro-apoptotic potential in colon cancer cells lines by activation of peroxisome proliferator-activated receptor gamma (PPRA-γ) [198,199]. Finally, experimental data showed overexpression of sodium-glucose cotransporter 2 (SGLT2) in colon cancer cells, and one study suggested beneficial effects of SGLT2 inhibitors in an animal model of CRC [200]. To the best of our knowledge, there is still no published data from clinical studies on therapeutic effects of particular GLP1-RA on CRC in T2DM patients. Nevertheless, two recent meta-analyses of randomized controlled trials demonstrated that the use of liraglutide, exenatide, and dulaglutide was not associated with an increased risk of malignancy [167,201]. Future studies are needed in order to reach a definitive conclusion on the role of these antidiabetic agents on CRC.

## 4. Conclusions

All the-above mentioned mechanisms: insulin resistance, hyperglycemia, oxidative stress, and inflammation, emphasize the interconnectivity and possible causality among DM and CRC. In light of these findings, both current and novel strategies for the treatment of DM should take into account the higher incidence of CRC in these patients and should be evaluated in terms of their possible beneficial effect in minimizing the risk for the onset of malignant disease. On the other side, antidiabetic medicines should be considered in CRC patients, having in mind a significant risk for developing diabetes in this population. 

In light of the above, there are no clinical studies that clearly indicate a negative effect of the use of GLP1-RAs in diabetic patients already suffering from colon cancer, in those under chemotherapy treatment, or those with a newly developed colon cancer. Therefore, it would be advisable in the case of diabetic patients to assess their past medical history and the occurrence of thyroid, pancreatic, and colic adverse events. The occurrence of colon cancer in parallel with GLP1-RAs use, compared to pancreatic and other glands’ cancer (especially thyroid), seems to be very low and, in any case, these treatments seem to reduce the possibility of cancer cell progression in subjects with a pre-existing cancerous lesion. However, since preclinical and clinical data are still not fully conclusive, caution is mandatory. Therefore, GLP1-RAs treatment in T2DM patients at high risk of colon cancer cannot be suggested according to the available evidence, and such therapy, if installed, must be carefully evaluated and monitored.

## Figures and Tables

**Table 1:** Clinical trials investigating the impact of antidiabetic therapy in T2DM patients on CRC risk or survival of patients with CRC.

Publication	Antidiabetic Therapy	N of Patients	Follow-Up	Results
Schiel et al. (2005) [194]	Insulin	147 patients with T2DM114 patients with T1DM	10 years	Increased incidence of colon and rectum cancer.
Home et al. (2010) [190] ADOPT trial	Metformin Rosiglitazone Glyburide	1454 patients with T2DM on metformin 1456 patients with T2DM on rosiglitazone 1441 patients with T2DM on glyburide	4 years	Number of patients who developed CRC in glyburide group was higher than in metformin and rosiglitazone groups. No obvious advantage of metformin or rosiglitazone.
Home et al. (2010) [190] RECORD trial	Sulfonylurea Metformin Rosiglitazone	1122 patients with T2DM on sulfonylurea and added metformin 1103 patients with T2DM on sulfonylurea and added rosiglitazone 1105 patients with T2DM on metformin and added sulfonylurea 1117 patients with T2DM on metformin and added rosiglitazone	5.5 years	Number of patients who developed gastrointestinal cancers was higher in the groups receiving sulfonylurea as background or add-on therapy.
Nauck et al. (2018) [191]	Liraglutide	4668 patients with T2DM on liraglutide 4672 patients with T2DM on placebo	3.8 years	No difference in colon or rectum cancer incidence in liraglutide and placebo group.
Singh et al. (2016) [192]	Metformin	115 patients with CRC and T2DM on adjuvant chemotherapy and metformin 152 patients with CRC and T2DM on adjuvant chemotherapy without use of metformin	6.5 years	No difference in disease-free survival, overall survival and time to recurrence between patients on metformin and without use of metformin.
Vernieri et al. (2019) [193]	Metformin	76 patients with CRC and T2DM on adjuvant chemotherapy and metformin 44 patients with CRC and T2DM on adjuvant chemotherapy without use of metformin	60.4 months	No difference in overall survival and relapse-free survival between patients on metformin and without use of metformin

## Data Availability

Not applicable.
